# Application of empathy theory in the study of the effectiveness and timeliness of information dissemination in regional public health events

**DOI:** 10.3389/fpubh.2024.1388552

**Published:** 2024-04-30

**Authors:** Min Zhang, Xiaobing Lu

**Affiliations:** ^1^Institute of Education, Nanjing University, Nanjing, Jiangsu, China; ^2^Department of Applied Foreign Language Studies, Nanjing University, Nanjing, Jiangsu, China

**Keywords:** public health events, empathy, information dissemination, empathy theory, public health emergencies

## Abstract

**Introduction:**

This study examines the role of empathy theory in enhancing the effectiveness and timeliness of information dissemination during regional public health events, with a focus on the SARS and COVID-19 pandemics as case studies. Utilizing an anthropological interview method, the research delves into the public's transformation from passive recipients to active participants in information dissemination, emphasizing the impact of empathy.

**Objective:**

The study aims to evaluate the application of empathy theory in the context of public health emergencies and to determine its influence on the quality of information dissemination and public engagement.

**Methods:**

The research involved two distinct surveys, each collecting 50 questionnaires from participants in different regions, to capture a diverse range of perspectives. The surveys assessed participants' views on information dissemination, their levels of empathy, and their behaviors in receiving and sharing health-related information.

**Results:**

The findings indicate that empathy plays a crucial role in facilitating the active involvement of the public in information dissemination. There is a notable difference in the public's emotional response and information sharing behaviors between regions with direct experience of the health events and those less affected.

**Conclusion:**

The study concludes that empathy theory, when applied to information dissemination during public health emergencies, can significantly improve the public's engagement and the overall effectiveness of communication strategies. The results underscore the need for empathetic communication to foster a sense of solidarity and collective action in response to public health crises.

## 1 Introduction

Today, we live in an era of rapid information dissemination, with various forms of information continuously being transmitted or received. In a regional public health emergency, the effectiveness and timeliness of information dissemination are particularly crucial as they directly impact the physical and mental wellbeing, even the safety, of people within the region (1). Information dissemination is not an event that occurs at a single point in time; rather, it involves a process composed of five elements: the sender, receiver, message, medium, and feedback. It's worth noting that the relationship between the sender and receiver is not fixed during the dissemination process. Therefore, information dissemination always carries a subjective perspective. Thus, from a theoretical standpoint, it is impossible to obtain completely objective information. Our goal is to present information as objectively as possible from others' perspectives, ensuring its effectiveness, objectivity, and timeliness throughout the dissemination process.

Empathy, originating from the field of psychology, refers to an individual's ability to understand and experience the feelings of others. Empathy encompasses both cognitive and affective dimensions. Cognitive empathy involves understanding the thoughts and feelings of others, while affective empathy requires individuals to experience the emotional states of others (2). In recent years, empathy theory has been widely applied in the fields of communication and interpersonal research. Scholars believe that empathy can enhance interpersonal understanding and improve communication effectiveness (3). In crisis communication, official accounts that demonstrate empathy are more likely to gain public trust. Empathy has also been applied in doctor-patient communication, helping to improve patient compliance (4).

Regional public health emergencies are characterized by their suddenness, uncertainty, and complexity, posing potential threats to public health. In this context, the effectiveness and timeliness of information dissemination are crucial for controlling the situation. Any disruptions in information transmission may lead to the escalation of the crisis and delayed rescue efforts. Moreover, transparent information disclosure can enhance public cooperation. Therefore, exploring how empathy theory influences the effectiveness and timeliness of information dissemination in regional public health emergencies will contribute to the formulation of crisis management and communication strategies. Existing research has found that empathy plays a significant role in crisis communication and public emotion regulation. While literature (5) has investigated the cognitive and emotional responses of the public to epidemic information based on empathy, there remains a notable gap in empirical studies focusing specifically on the application of empathy theory in improving the effectiveness and timeliness of information dissemination during regional public health events. Addressing this gap is essential for gaining a deeper understanding of the mechanisms through which empathy operates in such situations and for informing evidence-based communication strategies. Therefore, this paper aims to expand the application of empathy theory in this emerging field by employing a case study approach. Through the examination of diverse scenarios and contexts, this approach facilitates a nuanced understanding of the complex interplay between empathy, information dissemination, and public response in the face of regional public health crises. In regional public health emergencies, information dissemination often faces various challenges such as information overload, rampant rumors, and public panic, which to some extent trigger a crisis of trust in authoritative information sources (6). Effective information management is therefore essential, and empathy can serve as a coping mechanism.

The public often shapes collective cognition and emotions in the reporting of regional public health emergencies. News coverage of public health emergencies, especially those affecting specific regions, always plays a critical role in shaping local public emotions. For example, during the COVID-19 outbreak in Wuhan, China in 2020, news coverage of the event united the people of Wuhan in their fight against the epidemic, fostering a shared belief among most residents. Scholars generally believe that individuals with larger social support networks receive more social support and are better equipped to cope with difficulties and challenges. In today's information age, public opinion in the online environment evolves with various media news or reports, affecting public emotions as news evolves. The public in the online environment has transitioned from being merely “passive” to becoming “active” information consumers and generators. Empathy is a subset of emotions and is an innate human ability (7). Empathy is widely applied in the information dissemination of regional public health emergencies (8, 9).

This study aims to explore how empathy theory, initially proposed only in psychology, is applied to the information dissemination of regional public health emergencies from the perspective of empathy, and to investigate how people perceive the widespread application of empathy theory in the information dissemination of regional public health emergencies. Finally, we explore the impact of this theory on the effectiveness and timeliness of information dissemination, particularly in the context of regional public health emergency information dissemination.

## 2 The connotation and extension of empathy theory

### 2.1 Overview of empathy theory

The concept of empathy holds a central role in understanding human interaction and communication, particularly within the context of information dissemination during public health emergencies. However, the term itself carries some ambiguity. The popular understanding of “empathy” often refers to stepping outside of one's own perspective and experiences to understand another person's thoughts and feelings. This emotional resonance can lead to actions that benefit the other person, such as “standing in their shoes” to understand their situation.

Academically, the concept of empathy has a deeper history. German philosopher Robert Vischer first introduced the term “Einfühlunge” to describe the act of projecting one's own feelings onto external objects. Later, Edward Titchener proposed “empathy” as a replacement, defining it as “the process of humanizing the object, the process of feeling that we ourselves are inside something else”.

In Chinese translation, the term “empathy” presents a unique challenge. The concept of empathy remains contentious. It has several translations, including “empathy,” “sympathy,” “empathy,” and “empathy.” Chinese scholars have translated empathy as empathy, but they argue that this translation method fails to fully capture the word's complete meaning (10). The translation of “empathy” emphasizes its sensory aspect, while “Empathy” seems to consider both reason and feeling, with a focus on the rational aspect of human psychology. Translations can be categorized into three main types: the “Love” translation school, the “Sense” school, and the “Rational” translation. Despite the variations in translation, the core essence of empathy remains consistent. This paper defines empathy as the innate ability to directly perceive and comprehend another person's emotional state. It encompasses a multi-dimensional process that can be scrutinized from three primary viewpoints: attributes, essence, and organizational structure. (1) Attributes: this dimension delves into the capacity to directly sense and resonate with another's emotions. It investigates the biological and psychological underpinnings of empathy, exploring how human development shapes the ability to empathize with others. (2) Essence: this perspective interprets empathy as a cognitive manifestation. While valuable, it is constrained by its exclusive focus on the outcome of empathy, overlooking the intricate process involved. (3) Organizational structure: this dimension regards empathy as a multifaceted process characterized by various components. It delineates the stages of empathy, including active listening, perspective-taking, emotional response, and appropriate action.

**Figure 1:**
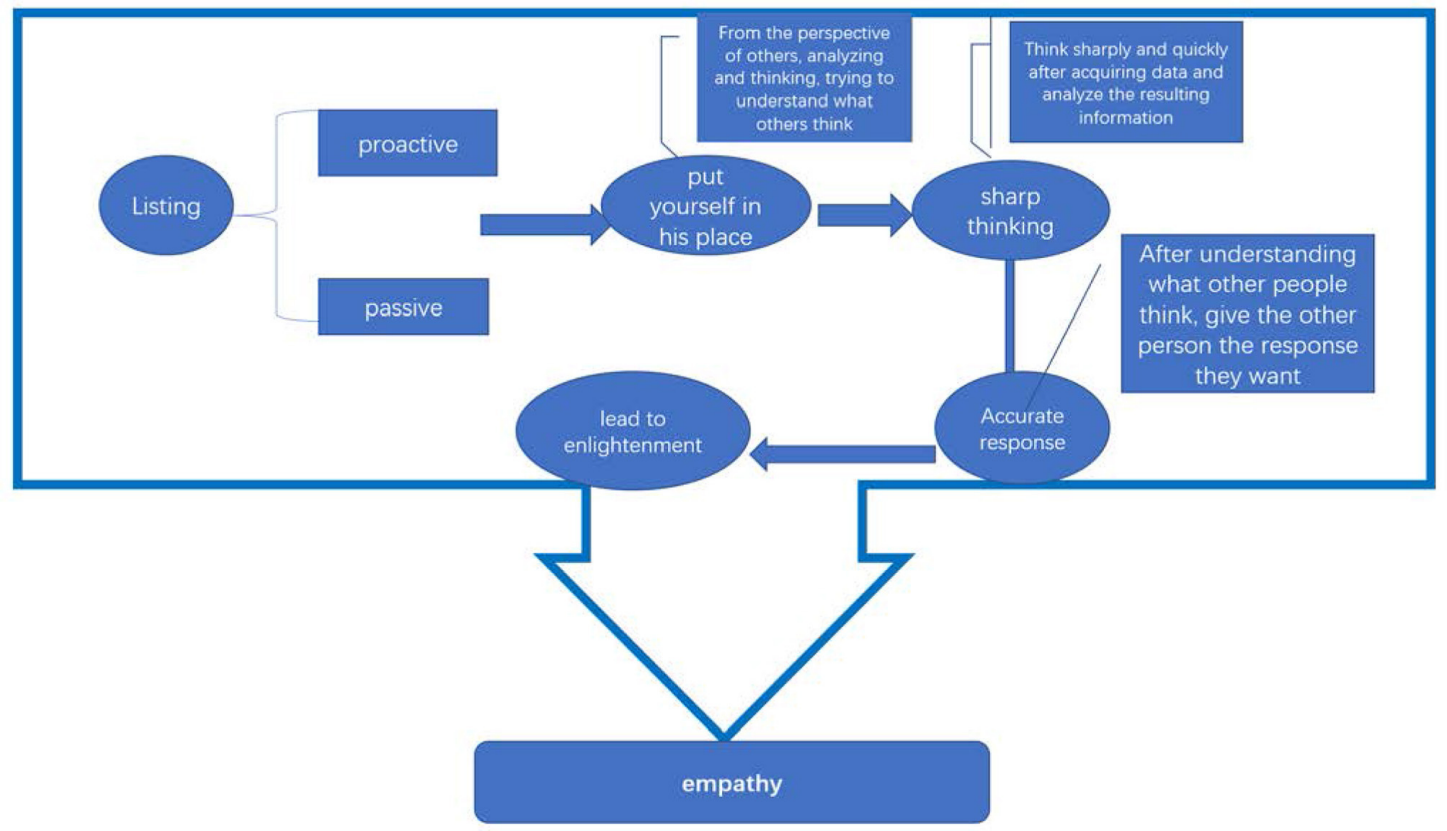
The process of empathy.

From the attribute dimension of empathy, empathy is the ability to directly emotionally feel the psychological situation or psychological state of the other, and to understand the emotions of others by feeling this state is universal. At the same time, the origin of empathy is the focus of this dimension of research, and the emotions formed by humans in the process of physiological and psychological development can directly feel what others feel. Starting from the essence of empathy as an entry point to analyze empathy, scholars usually regard empathy as a cognitive result, and this dimension as an entry point for the analysis of empathy has certain limitations, that is, only pay attention to the state and result of empathy but lack of empathy. The process that forms this is less studied. Finally, the research and analysis of empathy is carried out from the dimension of the organizational structure of the hectare as the entry point, and the advantage of this dimension research is that empathy has multiple components in the structure and has the characteristics of specific process, and psychological empathy is a continuous process of layer by layer. People often need to go through five stages: active listening, putting themselves in their shoes, thinking sharply, responding accurately, and leading enlightenment (Figure 1).

The view shared by most scholars in this process is that empathy includes three necessary components of the process and non-emotional outcomes of personal and situational factors that occur in empathizers (11).

### 2.2 Extension of empathy theory: empathy communication and the evolution of public opinion

#### 2.2.1 Empathy transmission

Empathy and communication seem to be intrinsically linked. Through the definition of the concept of empathy in the above content, it is not difficult to see that the formation of empathy is inseparable from the mutual communication and emotional sharing between people, so the connection between empathy and communication can be said to be closely related and inseparable. The process of studying empathy is also to a large extent also studying emotional cultural exchange and information dissemination. Today's concept of empathy communication is based on the concept of empathy. Empathy is now widely used in the field of psychology and anthropology, and the definition of empathy transmission is now not limited to individual-to-individual transmission, but also between individuals and groups, in the face of regional health events 2 The typical event of COVID-19 in Wuhan, China in 2020, is an example in which the public actively participates in empathy in the various emotional scenarios constructed in the face of COVID-19. This also breaks through the psychological theory of empathy, where empathy is limited to individual-to-individual connections.

The way of empathy transmission is through the diffusion of information after the dissemination and after sharing, it has an emotional resonance with a certain individual or group within a certain range, and thus produces similar emotions. Communication is often pursued by the communication effect, in communication science the communication effect is divided into cognition, emotion and attitude three levels. The conceptual definition of empathy corresponds to the second and third layers of the effect of communication. Communication activities can have a profound impact on people's views on social issues, and in terms of emotions, they can resonate with similar emotions or emotions, and they can also strengthen people's common will to have ideas or opinions about the same thing. The relationship between the effects of the three levels of the communication effect is complementary, and if the effect of one level changes, the changes at the other two levels will always occur.

#### 2.2.2 Evolution of public opinion

**Figure 2:**
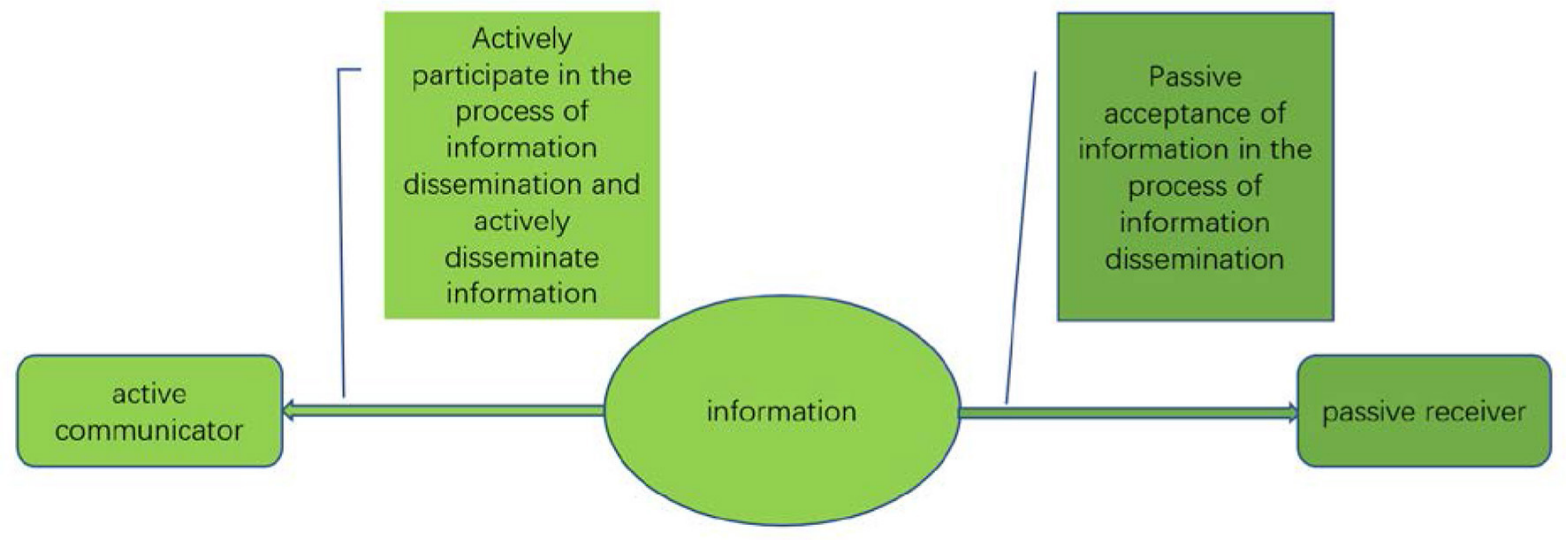
The transformation of mass identity in internet public opinion.

Compared with traditional social public opinion, the corresponding network public opinion shows different characteristics in terms of content generation methods, presentation methods and communication media. The so-called network public opinion refers to the collection of all attitudes, opinions, emotions and behavioral tendencies expressed by the public through the network platform about various matters related to their own interests or interests that arise in a specific time and space and are related to their own interests or interests of specific organizations or individuals. Network public opinion in public health events, including the main body of public opinion and the role of public opinion. Audiences, media, governments, and public opinion are themselves important subjects in dealing with online public opinion (12). The public is no longer a single communication audience or communication subject, and the public always participates in the process of network public opinion dissemination as a dual identity of communicator and audience (13). In this process, through the acceptance of information, transmission and transformation of information, its emotional consensus is quickly disseminated to the outside world (Figure 2).

After the COVID-19 epidemic has been reported by various media, it has quickly transformed into a public opinion issue on the Internet, and in this process, the groups participating in the discussion have their emotions and emotions communicating with each other, because they have the same or similar positions and emotions, so they are more likely to resonate. Just like Twitter users' content and usage attitudes, online public opinion information can be used to assess crisis situations and track and monitor disease conditions. This shows that empathy plays a crucial role in shaping online public opinion (14, 15).

## 3 The application of empathy in the dissemination of information in public health events

### 3.1 Regional public health events and network public opinion

Public health events are public measures taken by the public sector to prevent disease, increase life expectancy and improve health. These include not only public health events caused by non-pest control, but also public health events caused by epidemics, etc. Throughout human history, the world has faced four major “pandemics”, with the 1918 influenza pandemic being the deadliest, resulting in ~100 million deaths worldwide in just 2 years. At the same time, public health emergencies occur frequently, bringing about various online public opinion events (16). Due to the complex ecological environment of network information and the intertwining of the number of fake news, the resulting negative network public opinion is generated and spread rapidly, which creates a huge obstacle for public health departments to effectively identify, judge and formulate effective response strategies (17). Online public opinion events regarding public health emergencies are especially prevalent in the realm of social media. Since 2018, China has made it clear that public health incidents should be managed in the common good and in a model of coordinated governance by multiple entities and regions (18). Even so, there are many difficulties in responding to sudden regional public health events (19). For example, the dissemination of information is not timely, on the one hand, the lack of manpower and materials in a single region in responding to a public health event in the region makes it difficult to respond effectively to the event in the first place; On the other hand, due to the lack of timeliness and effectiveness of information dissemination, it is difficult for other regions to make correct judgments in the first place, thus affecting areas that help regional public health events. At the same time, even if the dissemination of information is timely enough, it is difficult to put oneself in the shoes of the incident and consider the problem in the place where the incident occurred, so that it is difficult to provide effective assistance in the case of assistance.

Online public opinion and empathy influence each other and promote each other. On the one hand, the development of online public opinion has a role in promoting the public's display of their own emotions, emotions and the behavior of expressing opinions, on the other hand, the participation of groups that generate empathy in the discussion of public opinion helps to enhance the influence of public opinion. Social networks have become the most important means of communication for sharing awareness, information and innovation. With the realization that even weak social relationships can have an impact, social networks have become a medium for marketing and influence-maximizing technologies (20).

### 3.2 Application of empathy theory in information dissemination

#### 3.2.1 Application of empathy in the SARS incident

**Figure 3:**
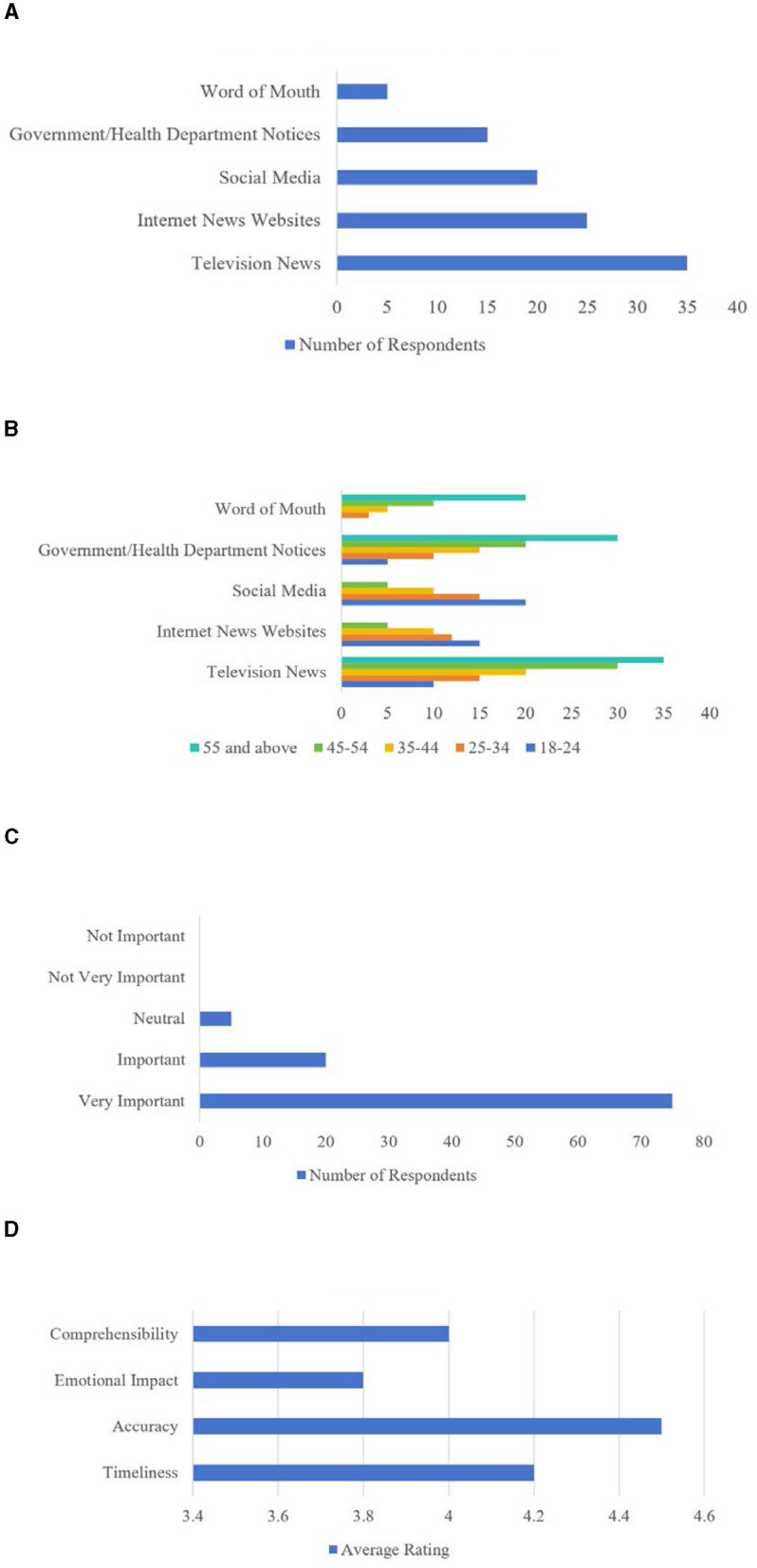
Statistics of survey questions. **(A)** Distribution of information channels. **(B)** Information preference among different age groups. **(C)** Evaluation of the importance of information dissemination by respondents. **(D)** Respondents' perceptions of various aspects of information dissemination.

With the frequent occurrence of natural disasters and disease transmission events in China in recent years, the subjective awareness of public participation in public health events is gradually improving, and with the continuous development of network information technology, smart phones are popularized nationwide, and the public is no longer a passive recipient of information dissemination in public health events, but gradually becomes an active participant in the dissemination of this type of event. In the transmission of cross-cultural empathy, the two sides come from different cultural backgrounds, and face differences, estrangements, and even conflicts and confrontations between heterogeneous cultures. It is true that in cross-cultural transmission, ethnocentristic or culturally centristic thinking such as “non-chinese species must be different in their hearts” and “Western-centrism” have always existed, which is the limitation of human beings derived from selfish genes. The spread of cross-cultural empathy guided by human beings is an important way to transcend this limitation (21). There are also a variety of ways to participate. Taking two of China's more famous public health events as an example, the first public health event that gave the whole of China a deep and heavy memory was the atypical pneumonia that spread rapidly to the whole country at the end of 2002 Event. At that time, this incident, because China lacked experience in dealing with such sudden and large-scale public health events, compared with today, the level of medical care was low and the materials were relatively scarce, and for the memory of that time, the vast majority of Chinese first thought of panic. The high mortality rate has left people in areas where outbreaks are more concentrated, such as Beijing, under the high pressure of the fight against the epidemic; However, for cities that have not been affected by the epidemic, such as Yinchuan in Ningxia, people do not have such a deep memory of SARS. There are many reasons for this phenomenon, one is because there is no local epidemic, people do not have a direct understanding of the things that have not happened around them, so the panic has not spread too much in the local area, and another important reason is because the information dissemination was relatively slow at that time, the channels were relatively small, and the people's reception of relevant information was limited and insufficient. The main reasons for this result are the underdevelopment of communication technology and the relatively low level of science and technology, for example, at that time, smart phones were not popularized throughout China, computers and the Internet were not popularized, and it can be said that China at that time had not yet entered the information age. People have fewer access to information, and can only learn about ephemeral and inadequate information through text messages from friends and family in the outbreak area or by phone. At this time, people participate in the process of disseminating information about public health events and act only as passive participants. Among the people who were involved in this incident, the author randomly selected 50 people as interview subjects in Beijing and Yinchuan, two representative cities, and used the research method of the interview method After collecting information and collating it, it was found that there was a big difference in people's understanding of the public health event of SARS in the two regions. We designed 50 questionnaires to explore the application of empathy theory in the dissemination of information during public health events. The questionnaire includes both quantitative and qualitative questions to collect data on respondents' perspectives on the dissemination of public health event information, their levels of empathy, and how they receive and share information through different channels. When designing the questionnaire, the questions were clear, unambiguous, and able to effectively measure the required data, as shown in Appendix 1. The composition of the 50 questionnaires in terms of personnel, age groups, and cultural levels is clear. To ensure the representativeness of the questionnaires, we sampled different populations, including various ages, genders, educational backgrounds, and occupations. A stratified random sampling method was used to ensure diversity in the sample's demographic characteristics. For respondents in Beijing and Yinchuan, the sample distribution of both locations was balanced to facilitate effective comparative analysis. The collected questionnaires were analyzed and summarized, with the results shown in Figure 3.

Figure 3A illustrates the main channels through which respondents acquire information on public health events and their distribution. Television news emerges as the most popular information channel, followed by internet news websites and social media. This reflects respondents' demand for authoritative and timely information. Figure 3B presents the preferred information channels of respondents categorized by age groups. Younger individuals (aged 18–24) tend to favor social media and internet news websites as sources of information, while older individuals (aged 55 and above) rely more on television news and word of mouth. This may be related to varying levels of familiarity and acceptance of technology among different age groups. Older individuals may be more accustomed to traditional information sources, whereas younger individuals are inclined toward digital and social channels. Figure 3C displays the distribution of respondents' evaluations of the importance of information dissemination for public health events. The majority of respondents (75%) consider information dissemination for public health events to be “Very Important”, indicating a widespread belief in the crucial role of timely and effective information dissemination in addressing public health crises. Additionally, 20% of respondents view information dissemination as “Important”, while 5% perceive it as “Neutral”. Notably, no respondents rated information dissemination as “Not Very Important” or “Not Important”, indicating a lack of negative attitudes toward information dissemination. Figure 3D presents respondents' evaluations and average ratings of different aspects of information dissemination for public health events. Respondents gave the highest rating (average rating of 4.5) to the accuracy of information, highlighting their emphasis on truthfulness and reliability. Timeliness and comprehensibility also received relatively high ratings, while emotional impact scored relatively lower. This suggests a need to consider how to evoke emotional resonance among audiences in information dissemination efforts.

Here, the author has selected two representative interview records to interview Mr. W from Yinchuan and Mr. Z from Beijing. The transcript of the interview is shown as Appendix 2.

**Figure 4:**
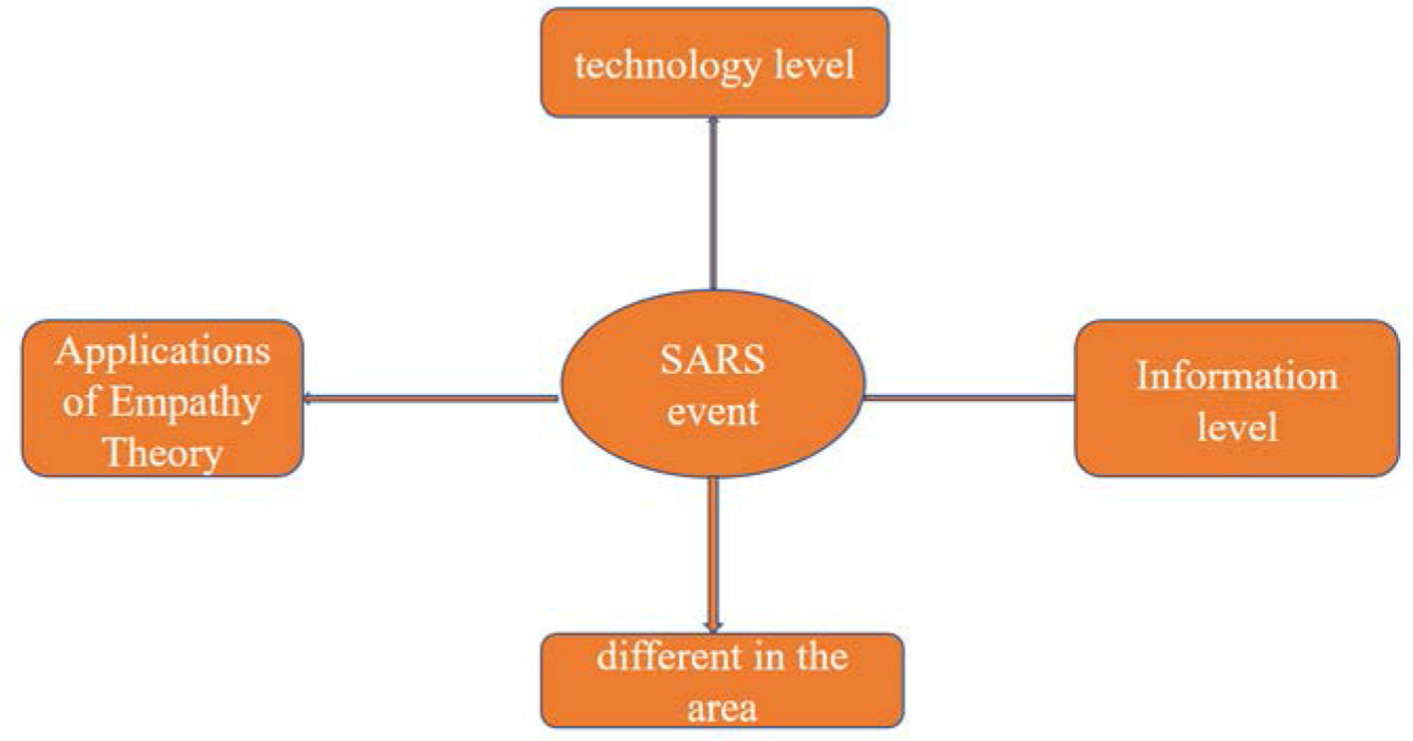
Factors affecting information dissemination of SARS incidents.

As show in Figure 4, through the above two typical interview records, it can be clearly seen that in the “SARS incident”, the dissemination of information has obvious deficiencies in timeliness and validity. At the same time, people in different regions have greatly different emotional and cognitive views of the same public health event. Even after nearly 20 years, the people who mention the event again can still feel the fear and fear of the heart, but in areas with less information and publicity and far away from the place where the event occurred, people's senses of the event are not so direct, and the impression is not as deep as in Beijing.

#### 3.2.2 Application of empathy in the COVID-19 incident

Taking the public health event of “COVID-19” in the past 2 years as an example, the author once again interviewed people in different regions in the form of interview method research, hoping to understand people's views on the same public health event through interviews. Among them, representative interview content was still selected, hoping to present the interviewee's views on the event as objectively as possible. The interview is shown as Appendix 3.

**Figure 5:**
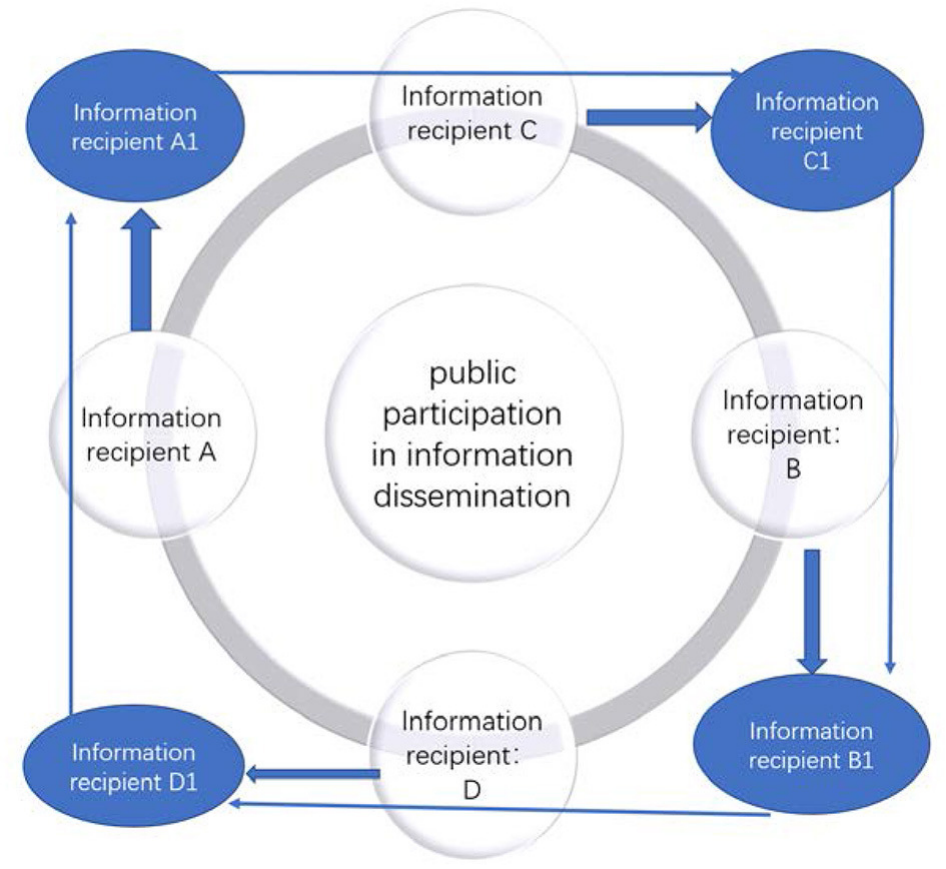
Information dissemination mode under the ripple effect.

A comparison of interviews from these two typical public health events clearly reveals significant differences in how people engage in disseminating information about the event and the extent to which they receive information. Among them, there are many factors that affect the dissemination of information, such as the level of media development, the different levels of scientific and technological development, and the degree of participation of the masses in information dissemination, all of which are related to whether information dissemination is effective and timely. According to the interview content of the two public health events, it is found that the level of science and technology and the degree of participation of the public in information dissemination are important factors affecting the quality of information dissemination. Leaving aside the factor of the level of science and technology, and discussing the degree of mass participation alone, empathy directly affects the degree of participation of the masses in the dissemination of information. Take this interview in Wuhan as an example, this interview obtained an important piece of information, compared to the news report this passive way of receiving information, people are more willing to participate in the dissemination of new media information, whether it is short video or short news, people can be more active according to their own needs to obtain timely and effective information, after obtaining information, a large number of people will make the information spread again through comments, discussions, and sharing. In this process, in view of the authenticity of the information, the masses also participate in the process of information dissemination to carry out individual screening, the multi-source dynamic characteristics of social media information make the user's independent analysis possible, a large number of users respond quickly to the content that does not conform to the facts, so that the false information can be effectively suppressed in the early stage of dissemination (22). Like the pattern of difference order proposed by Mr. Fei Xiaotong in his book “Native China”, he proposed that China's interpersonal relations have the characteristics of “difference order pattern”, forming a network of relatives and alienations that extend outward with self-centeredness (23). With a receiver of information as the center of the ripple, the information will continue to spread like a ripple, but the ripple will always return to calm due to the limitations of the limited system of a person's ability. However, if the recipient actively engages in discussing the information upon receipt and shares it with others, it's akin to casting a stone into the outer circle of ripples, thus enhancing the speed and effectiveness of information dissemination (Figure 5).

### 3.3 Application of empathy theory in information dissemination

The preceding discussion underscores the crucial necessity of applying empathy theory in disseminating information about public health events. However, it is not enough to effectively and reasonably apply empathy theory to the dissemination of information on such events and thus ensure the timely and effective dissemination of information solely by relying on the spontaneous participation of the masses. The author contends that implementing measures to apply empathy theory to the dissemination of information on regional public health events should begin with three main stakeholders. Firstly, there's a need for government support through policies, such as the enactment of relevant laws. Only in this way can we ensure the legitimacy of information dissemination on the one hand and effectively avoid the occurrence of some phenomena that hope to take advantage of the opportunity to spread illegal and false information to make profits for themselves. The second main body is various organizations, including news media platforms, enterprises and self-media teams. If the government plays the role of initiator in this process, then the organizations play the role of executor. The specific measures are to optimize and classify the information received by each platform by optimizing the communication channels, and promote the positive and event-related information, so as to minimize false information and a large number of meaningless confidence dissemination in the process of information dissemination. The third subject is the masses, and the spontaneous participation of the masses in the process of information dissemination can effectively ensure that the dissemination of information can be disseminated on a larger scale.

## 4 Conclusion

In conclusion, the analysis of 100 survey questionnaires, distributed across two distinct regions, has provided empirical evidence of the pivotal role empathy plays in information dissemination during public health events. The data revealed that in areas where individuals reported higher empathy levels, there was a marked increase in proactive information sharing and a more profound engagement with health communications. This pattern corroborates with the theoretical stance that empathy can be a driving force in communal response to health crises. The findings of this study not only affirm the theoretical propositions but also demonstrate their practical applicability in enhancing public health communication strategies. By integrating these insights, health communicators can craft messages that resonate on a deeper emotional level, thereby improving the overall effectiveness of information dissemination. This study paves the way for future research to delve into the nuanced impacts of cultural differences on empathy and its role in health communication across diverse global contexts.

## Author contributions

MZ: Conceptualization, Investigation, Software, Supervision, Writing – review & editing, Writing – original draft. XL: Data curation, Methodology, Writing – original draft, Writing – review & editing.
