# Supplementary material for: Application of empathy theory in the study of the effectiveness and timeliness of information dissemination in regional public health events
Source: Front Public Health. 2024 Apr 30;12:1388552. doi: 10.3389/fpubh.2024.1388552 (PMC11095106; doi:10.3389/fpubh.2024.1388552)
Supplement: Supplementary file 1 [file Table_1.docx]

**Appendix**

**Appendix-1**

**Questionnaire: Application of Empathy Theory in Public Health Emergency Information Dissemination**

| **Basic Information:** | |
| --- | --- |
| 1. Gender: | Male / Female / Other |
| 2. Age: | 18-24 / 25-34 / 35-44 / 45-54 / 55 and above |
| 3. Education Level: | High school or below / College / Bachelor's degree / Master's degree or above |
| 4. Occupation: | Student / Education / Healthcare / Government/Public Administration / Other (please specify): ___________ |
| **Perceptions of Public Health Emergency Information Dissemination:** | |
| 5. Importance of information dissemination during public health emergencies: | Very important / Important / Neutral / Not very important / Not important |
| 6. Channels used to obtain information during public health emergencies (Multiple choices): | Television news / Newspapers/magazines / Internet news websites / Social media (e.g., Weibo, WeChat) / Government/health department announcements / Word of mouth / Other (please specify): ___________ |
| **Assessment of Empathy Levels:** | |
| 7. Self-assessment of understanding others' emotions: | Very strong / Strong / Average / Weak / Very weak |
| 8. Ability to feel the emotions of victims in public health emergencies: | Always / Often / Sometimes / Rarely / Never |
| 9. Rating of statements based on feelings (1-5, where 1 means strongly disagree and 5 means strongly agree): | I can imagine the feelings of those affected in public health emergencies. / I pay attention to the stories and experiences of individuals in public health emergencies. / I tend to show sympathy for the victims in public health emergencies. / I feel I should take action to help those affected in public health emergencies. |
| **Information Reception and Sharing Behavior:** | |
| 10. Actively sharing information about public health emergencies: | Always / Often / Sometimes / Rarely / Never |
| 11. Main motivations for sharing information (Multiple choices): | To raise public awareness / To help others understand the situation / To express concern and support / To attract the attention of the government or relevant organizations / Other (please specify): ___________ |
| 12. Factors affecting willingness to share information during public health emergencies (Multiple choices): | Accuracy of the information / Emotional impact of the information / Influence of social and family members / Timeliness of the information / Other (please specify): ___________ |
| **Open-ended Questions:** | |
| 13. Suggestions to improve the effectiveness and timeliness of information dissemination during public health emergencies: | _______________________________________________________ |
| 14. Other suggestions or ideas: | _______________________________________________________ |

**Appendix-2**

| Q: Hello, do you have any impressions of the SARS incident, and can you describe how you feel about this incident after it broke out in China? And how did you learn about the event at that time?  W: For SARS, I can say that I was very impressed. At that time, I could obviously feel that there was still a great impact on daily life, I remember that all the primary and secondary schools in Yinchuan were on vacation at that time, and the classes were suspended for more than20 days, my wife and children were a little nervous at that time, I don't know what kind of disease this is, why is it so bad. We had to learn about the situation every day through television, mainly the news. At that time, the main thing was to watch the news broadcast, not as convenient as the current mobile phone Internet access, want to know what you do not have to deliberately look at, what Tencent news, WeChat, Douyin and other media in the official have (no) reports, that early (early in the morning) received the news. At that time, the mobile phone did not know how to access the Internet, and there was no computer at home. Then there is the chat with friends and colleagues when everyone chats about gossip that they don't know where they heard. At that time, the situation was what the news said, what we listened to, and then the neighborhood committee and the street notified the documents. However, these news sometimes wait for the notice to come down, it is no longer news, it should be "old news".  The same question was answered to Mr. Z from Beijing as follows: Regarding the SARS incident, I think that anyone in Beijing who experienced the fight against the epidemic at that time will be unforgettable. At that time, everyone did not know much about this disease, only that it was difficult to cure after it was learned, and this disease would be contagious, and it would kill people, when the epidemic broke out in Beijing, our reaction was first panic, because we did not know what the disease was, nor did we know how it spread in the crowd, to sum up is basically ignorant of it, people for unknown things, especially Things that can threaten human life are always full of fear. At that time, many hospitals in Beijing could not treat patients well because of insufficient medical standards and lack of medical equipment and materials, and finally the establishment of the Fang Cabin Hospital solved this problem to a certain extent. At that time, information was very scarce, and our information about "SARS" was mainly obtained through the news and some notices from the neighborhood committee, and sometimes when we received this information, it could not be regarded as new information. |
| --- |

**Appendix-3**

| Q: Hello, I believe that the "COVID-19" incident has a huge impact on you and the people around you, can you describe how you feel about this incident after the outbreak in China? And how did you learn about the event at that time?  Mr. L (Wuhan): The COVID-19 epidemic first broke out in Wuhan in our country, and I believe that everyone knows the situation of the epidemic in Wuhan. At that time, the epidemic in Wuhan attracted widespread attention from all over the country and even the world, and the blow of the epidemic to Wuhan was devastating, and the lockdown, isolation and death happened so closely for the first time. Especially for the first time, I felt that death was mercilessly taking away the lives of neighbors, relatives, and friends around me so closely. I'm lucky to be able to survive this outbreak. For the information of the epidemic, every day and all the time is received, news, vibrato, all kinds of APP software as long as there are information prompts on the mobile phone They are all pushing this information. Although isolated at home, I can receive useful information every day, of course, good and bad. What I feel most deeply by this information is that although we have locked down Wuhan, the people of the whole country have not abandoned us, and they have accompanied us in their own ways. Chinese people's hearts are tightly linked, and at this time, it seems that the epidemic is not so terrible.  Q: What do you think are the reasons why the dissemination of information is effective and timely during this public health incident?  Mr. L: One of the things that let me say is that because the level of science and technology has advanced, only mobile phones, the popularity of the Internet, although we are isolated at home, we can still use these high-tech products to obtain information. The second point is that most people are paying attention, forwarding, and commenting on relevant information. As long as the relevant information comes out, we don't have to find this information ourselves, the friends around us have a count, as long as it is a short video or news information, they will share what they think is useful to their friends and family for the first time. I think this is very important. Because only when more people participate in the discussion of the information, the dissemination of this information can be done faster, more accurately and more timely. |
| --- |
